# Complete Response to Sorafenib Rechallenge in a Patient with Metastatic Renal Cell Carcinoma

**DOI:** 10.1155/2017/2648471

**Published:** 2017-09-20

**Authors:** Ryo Kasahara, Noboru Nakaigawa, Kazuki Kobayashi

**Affiliations:** ^1^Department of Urology, Yokosuka Kyosai Hospital, Yokosuka, Japan; ^2^Department of Urology, Yokohama City University Hospital, Yokohama, Japan

## Abstract

A 79-year-old Japanese man underwent a medical examination for hoarseness. Computed tomography revealed a left renal tumor, and radical nephrectomy was performed. The tumor was a clear cell carcinoma. Fourteen months after the operation, the tumor had metastasized to the spleen, right lung, and retroperitoneal lymph nodes. We initiated molecular targeted therapy sequentially with sorafenib, sunitinib, and axitinib and then conducted a rechallenge with sorafenib. His metastatic lesions had completely vanished 5 months after initiation of the rechallenge. Ten months after the rechallenge, lumbar vertebral body metastasis appeared. However, we consider that the sorafenib rechallenge was effective because of the very slow growth of the metastatic lesion, with no other metastasis for 30 months, at the time of writing this report. Approximately 7 years after the first local recurrence, he remained alive, with relatively normal daily functioning.

## 1. Introduction

Renal cell carcinoma represents 2-3% of adult malignancies [1]. Patients with metastatic renal cell carcinoma (mRCC) have particularly poor prognoses, with 5-year overall survival (OS) rates <10% [2]. Recently, molecular targeted therapies such as tyrosine kinase inhibitors (TKI) and mammalian target of rapamycin (mTOR) inhibitors for treating mRCC have improved the prognosis of patients with mRCC and are effective for prolonging OS [3, 4]. For sequential therapy, we sometimes consider rechallenge therapy, during which one agent is administered again after it was discontinued for disease progression. We encountered a case of complete response with sorafenib rechallenge therapy. To the best of our knowledge, the present case is the first of its kind to be published.

## 2. Case Presentation

A 79-year-old Japanese man with a previous history of tuberculosis, myocardial infarction, cerebral infarction, diabetes mellitus, and hypertension noticed hoarseness in December 2008. He underwent a medical examination at the hospital and whole-body positron emission tomography-computed tomography (PET-CT) was performed, showing a left renal tumor. The patient was transferred to our department.

The left renal tumor was clinically diagnosed as renal cell carcinoma. Left nephrectomy was performed, and the pathologic diagnosis was clear cell carcinoma (pathological T3aN0).

**Figure 1:**
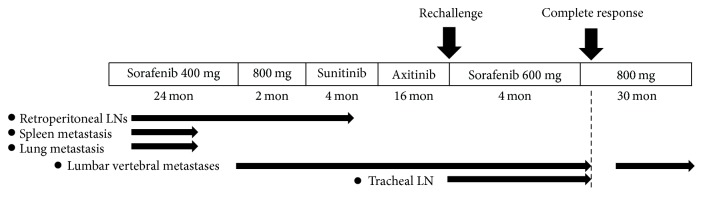
Sequence of events for initiation of molecular targeted therapy and for appearance of metastatic lesions.

Six months after the operation, follow-up CT revealed local recurrence (14 mm). However, upon evaluation of the patient's age and activities of daily living, we opted for observation every 2 or 3 months using PET-CT. Fourteen months after the nephrectomy, PET-CT revealed metastases to the spleen, right lung, and retroperitoneal lymph nodes (LNs). We initiated molecular targeted therapy with sorafenib (400 mg/day). We did not increase sorafenib dosage owing to adverse reactions including malaise and hand-foot syndrome. However, the treatment was effective, as evidenced by shrinkage of the metastases on PET-CT. He continued the therapy, and the majority of metastases disappeared, except for those at the retroperitoneal LNs. However, 24 months after the sorafenib initiation, new lumbar vertebral body metastases appeared. We subsequently increased the sorafenib dose to 800 mg/day (Figure 1).

We discontinued sorafenib administration 26 months after it was initiated, owing to obvious growth of the metastasis and increased maximum standard uptake value (SUVmax) of the lesions. Next, we initiated therapy with sunitinib at 37.5 mg/day. Sunitinib administration was stopped after 4 months because tumor expansion was evident on PET-CT. The patient was not treated with bisphosphonate agents or anti-RANKL antibodies, as he had tooth pain, and his lumbar pain was mild. Furthermore, irradiation therapy (to target bone metastases) was not administered.

As third line therapy, we started axitinib administration at 10 mg and increased the dose weekly, while monitoring for adverse effects. With axitinib, the tumors were effectively controlled for 16 months.

**Figure 2:**
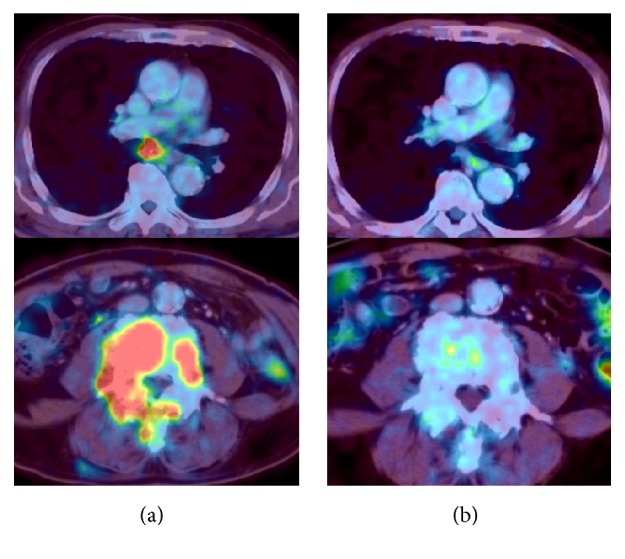
PET-CT showing (a) lymph node metastasis near the bifurcation of trachea and lumbar vertebral metastasis before sorafenib rechallenge therapy and (b) that the metastatic lesion vanished 5 months after the rechallenge therapy.

However, PET-CT revealed new lymph node metastasis near the tracheal bifurcation (Figure 2(a)). We decided to start sorafenib rechallenge therapy at 600 mg/day. He was diagnosed with stable disease 4 months after the initiation of the sorafenib rechallenge. Because there were no severe adverse effects, we increased the sorafenib dose to 800 mg/day.

In the next month (5 months after the sorafenib rechallenge), PET-CT showed dramatic shrinkage of the metastatic lesions, considered a complete response (Figure 2(b)). Ten months later, PET-CT revealed lumbar vertebral body (L3) metastasis. However, this lesion was growing very slowly; therefore, we continued sorafenib administration. This sorafenib rechallenge therapy effectively suppressed the tumor for approximately 30 months, and at the time of this report, the patient was alive for 7 years after the first local recurrence.

## 3. Discussion

In Japan, as of October 2016, four TKIs (sorafenib, pazopanib, sunitinib, and axitinib) were available for mRCC treatment. mTOR inhibitors (everolimus and temsirolimus) and nivolumab, which is an immune checkpoint inhibitor that recently became available, can also be used.

Although these molecular targeted agents improve the survival duration for mRCC [3, 4], almost all cases of mRCC progress at some point due to multiple mechanisms of resistance to the drugs. Sequential therapy using alternative TKI agents, mTOR inhibitors, or nivolumab is the current standard therapy after the appearance of severe adverse effects or development of resistance to first-line molecular targeted therapy in mRCC. By using several agents in an appropriate order, overall progression-free survival (PFS) can be extended by up to 27 months, subsequently improving OS for up to 40 months [3].

The efficacy of traditional anticancer drugs that fail to suppress tumor proliferation is never restored. However, with molecular targeted therapy for mRCC, the tumor can be suppressed by rechallenging (reusing) one drug that previously failed.

Nozawa et al. used sorafenib as rechallenge therapy for 12 patients with mRCC. While 8 patients achieved stable disease, none of the patients achieved partial or complete response [5]. In the same study, the median PFS with sorafenib rechallenge therapy was 5.4 months. The PFS with rechallenge for the present case is considered relatively longer. Furthermore, complete response with sorafenib rechallenge therapy has not been reported previously.

In the present case, response to the sorafenib rechallenge was not achieved at 600 mg/day, but complete response was achieved with a dose increase to 800 mg/day. Consequently, when the tumor is not controlled with a rechallenge, it may be possible to increase the dose of the agent, with careful attention to adverse effects.

The mechanism of rechallenge therapy is unknown, but it may provide an additional treatment option for patients who have already undergone sequential therapies. Therefore, we should consider rechallenge therapy in sequential therapy.
